# Education for the translation of Advanced Therapy Medicinal Products

**DOI:** 10.3389/fmed.2023.1125892

**Published:** 2023-04-04

**Authors:** Davide Adamo, Eustachio Attico, Graziella Pellegrini

**Affiliations:** ^1^Centre for Regenerative Medicine “Stefano Ferrari”, Interdepartmental Center for Stem Cells and Regenerative Medicine (CIDSTEM), University of Modena and Reggio Emilia, Modena, Italy; ^2^Holostem Terapie Avanzate s.r.l., Modena, Italy

**Keywords:** ATMP, multidisciplinary, regenerative medicine, translational medicine, valley of death, gene therapy, cell therapy, education

## 1. Introduction

New treatments at the forefront of personalized medicine are distinguishable from other treatments by some huge advantages over standard medicinal products. Cell and gene therapy, for example, act through multiple sophisticated biological mechanisms to replenish tissue, repair damages, and restore the microenvironment. This is possible through specific physiologic autocrine and paracrine functions, sometimes producing immunomodulation or stable integration of cells or correction of the genetic disorder, with the ability to sustain continuous therapeutic effects. Successful examples include (i) gene therapy to treat adults and children with loss of vision due to inherited retinal dystrophy by *in vivo* administration of adeno-associated viral vector (AAV) (1); (ii) gene therapy for adenosine deaminase (ADA) deficiency, whereby the lives of immunodepressed “bubble-children” can be saved *via* the permanent restoration of the missing enzymatic function (2); (iii) tissue engineering of the ocular surface, with the restoration of functional stem cells in limbal stem cell deficiency (LSCD) related blindness (3); (iv) the early stage therapy of epidermolysis bullosa in which the genetically modified epidermis can maintain the adhesion and functions of the patient's skin (4); (v) the use of embryonic stem cells to treat blindness due to macular degeneration (5); and (vi) chimeric antigen receptor (CAR)-T cells promising therapeutic approaches for treatment of pediatric and young adult refractory hematologic B-cell malignancies as well as adult relapsed or refractory large B-cell lymphomas (6). Regarding the CAR-T cell therapies, after the first two products approved in 2017 (i.e., Kymriah^®^ and Yescarta^®^) we reached a number of six CAR-T cells released in USA and European Union (EU) up to 2022 (7).

In the EU, these therapies are referred to as Advanced Therapy Medicinal Products (ATMPs), and they are currently regulated by the European Medicines Agency (EMA) as standard medicinal products (8). The need for pharmaceutical rules emerged from the awareness that new biologic therapies had been and were being developed for human treatment, some of which had already been used in the clinical setting for 10 years before the implementation of the regulations in 2007. These included tissue engineering of the ocular surface and gene therapy for ADA deficiency.

ATMPs comprise one of the most complex organizational and regulatory sectors that currently exist in clinical research. They are composed of living cells being or not genetically modified in contrast to stable chemical compounds and are distinguished by their variability in human donor genotypes with or without genetic modification. Because of this complexity, many ATMPs are lost in the so-called “valley of death” of translation from the laboratory bench to the bedside (9). An example is the California Institute of Regenerative Medicine, which up to 2019, spent more than 3 billion US dollars on research in stem cells, achieving only one clinical application. This clearly shows how difficult it is to efficiently translate research to patients when this is done with the highest research standards (10).

Fortunately, despite the hardship encountered, there are also successful examples. One of several cases is Holoclar^®^, the ATMP used for a form of blindness called LSCD, which is the transplantation of autologous cultured limbal stem cells. This technology was first published in 1997 (3), and before EU regulatory enforcement [Regulation (EC) No. 1394/2007], it had already been applied to 219 patients, guiding the translational process (11, 12).

It is worth noting that the shift from this first successful clinical application toward full compliance with the European GMPs (good manufacturing practices) regulation could not be easy, and any newly introduced difference could increase the variability of the whole process and decrease therapy standardization, as occurs for other products (9). Such changes might include, for example, the replacing of research reagents used in the development with clinical-grade ones and the introduction of specification limits for any manufacturing parameter or equipment.

## 2. The landscapes for new ATMP development

Generally, scientists feel overwhelmed by the endless regulatory obligations, resulting in three types of translation scenarios (13): (i) Scientists try to translate ATMPs into clinical practice using a do-it-yourself approach based on good scientific foundations but insufficient regulatory knowledge (Academy developing ATMPs); (ii) Scientists rely on big pharmaceutical companies, which have strong expertise in regulation but insufficient knowledge on the biology of specific cells, crucial for the development of the related ATMP (Companies acquiring ATMP licenses); and (iii) Scientists develop such therapies outside the regulated environment (Unproven therapies).

### 2.1. Academy developing ATMPs

The first described scenario occurs when the original technology (3) is progressively modified to meet regulatory standards. This implies a costly, reiterative process as some changes result in different technological robustness, or efficacy, with the risk of product rejection. Overall, difficulties can be:

• Inconsistencies between regulatory authorities' advice and biological mechanism of the ATMPs: the biggest challenge is to preserve the drug activity (i.e., potency of the product) while scaling up the manufacturing process and implementing the newly requested validations. Several significant modifications of raw materials, manufacturing methods, and controls should be introduced and personnel specifically trained (11, 14).
• Changes in clinical protocols to generate regulatory grade clinical data: regulatory authorities ask for protocol modifications without deep knowledge of the biology of the specific system. Indeed, not all approaches are suitable for different treatments, i.e., “one size does not fit all”; therefore, diverse solutions are needed for many regenerative medicine procedures.
• Safety considerations and safety monitoring: some required safety-related changes can rather result in reduced safety or efficacy of the ATMP. For example, the removal of serum or feeder layer in Holoclar^®^ ATMP result in a dramatic decrease in the percentage of the desired stem cells, which entails alternative approaches to guarantee the safety and efficacy of the process. Indeed, previous findings highlighted that epithelial cultures are optimized to contain a physiologic number of stem cells needed to restore the original physiology of a patient's tissue (15, 16). In-depth investigations of the impact of requested modifications under the selected conditions are always needed.

Previous experiences on the expansion of keratinocytes for the treatment of massive life-threatening full-thickness skin burns (17) revealed that the control of the different variables was instrumental in the timely treatment of patients to keep them alive. The previous practices are very helpful in offering creative solutions to meet some standardizations while maintaining the flexibility needed to manage living cells and tissues.

The related difficulties in complying with European GMP regulations regarding manufacturing, controls, and clinical application can complicate planning, increase costs, and result in a low recovery of investments with a consequent decrease in investor confidence. Moreover, the reiterative “do-and-redo” process related to regulatory issues and the unrealistic timing and costs for approval also conflicts with the urgency of patient expectations.

### 2.2. Companies acquiring ATMP licenses

The second scenario can occur when different professionals from pharmaceutical companies are involved in the regulatory compliance, clinical planning, and finalization of the ATMP project. Their involvement give rise to more standardized regulatory planning, but they often have limited knowledge of the impact of variations in the specific manufacturing process and clinical treatment.

This condition also slows down the preclinical and clinical programs since such studies should be customized around the product-specific features. This scenario was described for several products (18), whereby the records of the manufacturing facility experiences were inconsistent with the clinical design, chemical manufacturing and control (CMC) characterization, and preclinical studies due to fragmentation of the expertise in the different areas.

### 2.3. Unproven therapies

The common denominators in both the first and second scenarios are a lack of knowledge and an inconsistency between the required areas of competencies. However, while struggling with the regulations and maintenance of drug activity, some authors also faced the third scenario. Despite the regulatory rules concerning ATMPs being well established and considered to be relatively strict, their implementation differs among EU member states. Additionally, the lack of sufficient regulations in certain EU countries creates a danger of intra-European “stem cell tourism” (19, 20). Unfortunately, despite clear lessons from different countries, some regulatory authorities and regulators are, in fact, tolerant of unorthodox offers of non-certified therapies proposed as minimal manipulations (21). In addition, many clinics worldwide administer unproven stem cell therapies to patients with devastating diseases, often in the absence of any scientific rationale (22). This was highlighted in the recent years when the COVID-19 pandemic has increased the proposal of self-proclaimed treatments including more appealing stem cells-like based approaches (23, 24). Indeed, the advertisement of commercial entities of unproven therapies on their websites is especially cheeky. For the non-specialist public it is complex to realize that those are not justified cures as companies offering such treatments use several solid appearing arguments. However, and unbeknown to the public, these arguments are derived from other then a scientific and medical point of view (25, 26). In addition, simple registration of a clinical trial at the www.clinicaltrials.gov website is considered the equivalent of proof of therapy, with some companies using this website as a means of advertisement (27). Meanwhile, the trials listed on this website are not verified before posting, and numerous clinicians should recognize this uncertainty. Also, some treatments, defined as therapeutic alternatives, have been applied to different patient selections or disease severity, devoid of any in-process controls.

### 2.4. Demonstrating the safety and efficacy of ATMPs

Although complex, the “valley” between stem cell science and therapies is certainly bridgeable. The problems and divergences discussed above apparently emerged along with the enforcement of regulations and had an impact on industrialization. Obviously, the regulations were and are aimed at verifying the safety of procedures before human application; indeed, many ATMPs are composed of living cells, and they can thus be inherently risky due to the requirement of cell stimulation and characterization. However, safety is not sufficient as a parameter for their approval: the efficacy represents a crucial aspect because invasive procedures are often required for ATMPs application. Hence, the formal, unambiguous demonstration of their efficacy is mandatory before any registration or formal authorization. For instance, commercial clinics offer unproven adult “stem” cell “therapies” claimed to exhibit unique stem cell properties and exert numerous types of therapeutic activities. Interestingly, even advertisements frequently do not stress the direct regenerative properties of such cells. For example, experimental and clinical trials demonstrated that injection of “mesenchymal stem cells” (MSC)—often improperly considered universal stem cells—resulted in minimal engraftment (28–30). That's why the salvage concept of the paracrine effect became particularly popular [for review, see Langrzyk et al., (31)]. However, any cell can exert the paracrine effect; this does not automatically mean that any cell can be considered a curative stem cell—if their mechanisms of action are not linked to regeneration processes—and that they can exert therapeutic effects. This scenario can worsen when the patients are charged with these unproven therapies, also creating the risk of generating false-positive data. Indeed, when patients are required to pay for therapy and its effectiveness is assessed in an observational way using subjective criteria, the risk of creating a placebo effect and reporting false positive outcomes increases.

Finally, and most importantly, the serious side effects arising from those unjustified therapies may bring fear and resistance against stem cell therapies in the general public, thus preventing the development of rational approaches based on therapeutic stem cells and/or cells differentiated from them.

Reasonably, more focus is needed on cross-education of biotechnologists/scientists and regulators to improve safety, efficacy and to maintain the risk/benefit balance properly.

## 3. Education can bridge the gap between stem cell science and therapies

### 3.1. To become professional

In many fields, particularly in high-quality technologies, the starting point of any good practice is the professionalism (i.e., proficiency) of the personnel setting up the process. Professionalism is founded on accurate training and experience on a specific topic, as required in GMP rules. It seems obvious, for example, to hire an expert heart-surgeon to perform cardiac surgery; however, professional education for the development and production of ATMPs has never been properly formalized. This path requires experts to have comprehensive knowledge in at least two fields: the biology of specific tissues and the understanding of regulatory rules for selecting appropriate manufacturing and control conditions. In the progression of ATMP development, a deep understanding of the pathology to be treated and the related surgical approaches becomes mandatory.

The first gene and cell therapies approval experiences highlighted that a new generation of biotechnologists is needed and that their training, to become expert professionals in this field, would involve an understanding of regulators' language. This would help the communication and interaction with regulatory authorities in routine practice. Finally, early collaboration with physicians and surgeons enable a fluent and consistent translation from the preclinical to the clinical application of the ATMP. In some Institutions, this training is now initiated from the University education to convey the planning of ATMP development from multiple perspectives.

### 3.2. Regulatory biology co-education

Regulatory education was included to encourage professionalization during the progression of ATMP, to limit the time and costs involved in therapy development, as well as to identify problems early on. This step, which should be propaedeutic to any therapy planning (9), instilled an appropriate understanding and knowledgebase for the selection of safe culture conditions, raw materials, scaffolds/carriers, gene correction and validation methods. It is useful to include not only theoretical instruction, but also practical training exercises held under real conditions in GMP facilities, including all professionals involved in translational work. More importantly, this knowledge enabled an appropriate dialogue between specialists and regulators for concerted efforts to find congruous solutions regarding safety and process robustness. In the Universities, the presence of Regulatory Offices could support this dialogue since the first steps of the academic ATMP development.

Finally, the ATMP planning phase should include a productive early dialogue between scientists and statisticians to interpret findings from trials correctly. For instance, a Statistical Analysis Plan (SAP) could be designed and tailored to the specific ATMP.

### 3.3. Early biological-medical interaction

The interaction and reciprocal understanding of scientists and medical doctors are imperative and can trigger bidirectional learning. Biological research disconnected from clinical realities can convert optimal basic science into inefficient clinical results.

The first goal of ATMP developments is the identification of the medical need. The selection of appropriate medical needs is a critical requirement for establishing the importance of research and achieving its aims. Certainly, the development of complex and expensive therapies is justified only when standard therapies have defined limitations, are unavailable, or are associated with adverse events, low success rates, or high recurrence rates. Therefore, only in these conditions a certain amount of time, risk, or uncertainty can be taken to solve a clinically relevant need. Financial investments are offered when a real prospect of success-related reimbursement exists once the therapy is approved.

In our experience on the development of the first EU approved stem cell-based product, the early biotechnologist-surgeon interaction facilitated the understanding of the therapy for coordinated efforts to support an appropriate product definition. This involved the identification of conditions for *in vivo* integration in the specific pathologic microenvironment and then the associated drug selection, type of surgery, and outcome definition at specific time points of follow-up. Moreover, this interaction is instrumental in the definition of an accurate patient selection by use of biological markers and the development of new methods by sharing knowledge, as also highlighted by Deng et al. (32).

From a commercial point of view, surgeons are considered to be clients; however, above all, they are suppliers of the starting material for the whole process. This implies that regulatory authorities request for them official quality training on clinical procedures, from biopsy to follow-up. Such training can reduce the variability between surgeons, physicians, and nurses engaged in all steps of the clinical trials and later in the widespread application of the therapy.

### 3.4. The importance of long-term follow-ups and dissemination

The collection of any ATMP data is critical to reduce the rate of failure or late adverse events and is of utmost importance for scientific, regulatory, ethical, and business reasons. This holds true for several successful examples of advanced therapies (2, 16).

In our experience, after the clinical application, scientists and clinicians periodically share their observations and analyze the follow-up data over a very long term. This practice frequently improves the understanding of the real mechanisms of action of the ATMP since human physiology and molecular peculiarity can produce significantly different results from those of the animal models. Adverse events provide useful information and should be carefully taken into account. For example, some documented failures when analyzed enable the selection of appropriate specification limits for the potency marker of the therapy (as TP63 protein in Holoclar^®^) with the definition of the minimal amount of required stem cells for a successful corneal lifelong renewal (16, 33).

Continued patient observation can be costly in terms of time and resources but can have unanticipated, positive regulatory, and business benefits. Aside from decisions regarding the continuation or cessation of the therapy, possibly driven by perceived side effects and benefits, the evaluation of the recovered quality of life can have an impact on the regulatory approval of the product and reimbursement of the therapy. This approach, and the shared experience with worldwide experts, have also supported new classifications and grading of pathologies by defining patient subtypes. This resulted in an update of the selection criteria, medical knowledge, business aspects, and approval of the product (22).

The huge amount of data collected during development, clinical application and follow-ups of the ATMPs could be easily and smartly stored in a proper online register. This could serve as a useful database to be readily analyzed by big data experts in order to infer peculiar information.

Finally, sharing knowledge, including negative results, can avoid the reiteration of errors by different research groups, the waste of public and private money, as well as patient suffering associated with the loss of confidence.

### 3.5. What did we learn from our experience?

The evolution of this field and the multiplicity of approaches for the development of new therapies require regulatory tailoring of the new technologies. On this basis, negotiation and dialogue with competent authorities are only feasible and efficient after a common language is developed through the multidisciplinary education of professionals. Students' qualification and practical experience in the new background are critical to ensuring the quality and continuity of these projects. The staff becomes able to develop their visions, mindful of the experiences of other groups, with an increased capacity for the optimization of manufacturing processes, which is also associated with opportunities for professional development. Finally, prior knowledge and the effect of on-the-job training reduce the risk of GMP burnout during or after the training, saving time and resources.

## 4. Conclusions

Regenerative medicine represents one of the most promising hi-tech industrial areas of the future and has relevant growth potential in the medium- to long-term. Important results have been achieved in recent years (34), but much more can still be done to transform this activity into a substantial engine for European economic growth and competitiveness (35).

**Figure 1:**
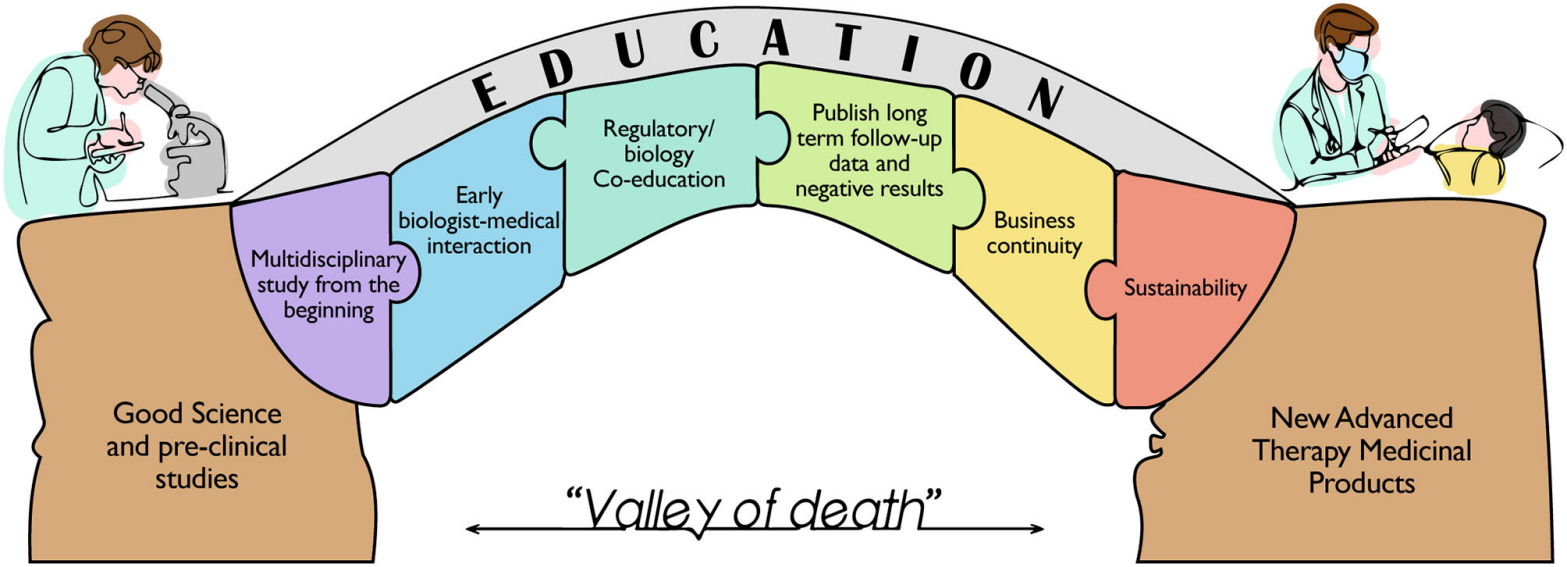
Schematic representation of the milestones needed to bridge the gap between the huge number of preclinical studies based on solid scientific evidence and the very limited number of Advanced Therapy Medicinal Products currently available for the patient's care. The figure highlights the building blocks for the education of a new category of professionals that can boost an efficient translation from bench to bedside, overcoming the so called “Valley of death”.

The intention to bridge the gap between basic stem cell science and therapies is complicated by the wide interpretation of translation, as well as the need for multidisciplinary teams and training. The past experiences revealed that the biggest issues arose after non-tailored pharmaceutical regulatory rule implementation, despite the need for rules to ensure the safety of therapies for the public (36). This improvement was not firstly followed by the appropriate education of professionals to build skilled and multidisciplinary teams with the ability to collaborate for the development of a complex medicinal product and bring it to approval as an available therapy (Figure 1).

The appropriate management of the translation phase was critical for the authors, as failures can not only lead to the development of an ineffective product but may also kill promising therapies.

Patient organizations also play a very important role in ensuring continuous feedback and communication among the different stakeholders, which in turn are instrumental for success.

Indeed, we believe that marketing authorization is not the final goal for successful advanced therapy. A therapy, once established and certified, should be available and regularly applied to patients who need it to be publicly valuable. In the last years, we are witnessing the withdrawal from the market of many ATMPs that were not profitable enough for the companies selling them (37–39). These therapies—associated with high production costs—aim to treat rare diseases with a limited number of patients who can be treated worldwide. This scenario leads to a paradoxical situation in which, although highly personalized and advanced therapies are known to cure devastating diseases, they are unavailable to patients who cannot benefit from them. The companies are free to decide their policies; however, after the repeated lack of consideration for the suffering of patients and their relatives associated with decades of research and public funding efforts to achieve reliable treatments, the politics and governments should follow up the previous investments with new approaches aiming to the long-term benefits for the public healthcare and, above all, for the welfare of the sufferers.

In conclusion, this paper, inspired by some past experiences, highlights the importance of comprehensive early education and cross-talking of all professionals involved in the ATMPs translational process, learning from the path that brought some ATMPs to be authorized for the market, highlighting the educational process.

## Author contributions

Conceptualization, writing—original draft preparation, supervision, and funding: GP. Resources: DA and EA. Writing—draft: DA, EA, and GP. All authors have read and agreed to the published version of the manuscript.
